# Di-μ-chlorido-bis­[aqua­(2,2′-bipyridine-κ^2^ *N*,*N*′)chloridocobalt(II)]

**DOI:** 10.1107/S1600536809027846

**Published:** 2009-07-25

**Authors:** Li-Li Zhu, Yu Sun, Huai-Hong Zhang, Yun Wang, Bai-Wang Sun

**Affiliations:** a Department of Chemistry, Key Laboratory of Medicinal Chemistry for Natural Resources, Ministry of Education, Yunnan University, Kunming 650091, People’s Republic of China; b College of Pharmacy, Jiangsu University, Zhenjiang 212013, People’s Republic of China; c Ordered Matter Science Research Center, College of Chemistry and Chemical Engineering, Southeast University, Nanjing 210096, People’s Republic of China

## Abstract

The title complex, [Co_2_Cl_4_(C_10_H_8_N_2_)_2_(H_2_O)_2_], is composed of two Co^II^ atoms, each hexa­coordinated by three Cl atoms, one 2,2′-bipyridine (bpy) ligand and one water mol­ecule in a distorted octa­hedral geometry. Neighboring Co^II^ atoms are linked together by two Cl bridges, forming a dinuclear Co^II^ complex with inversion symmetry. There are inter­molecular O—H⋯Cl hydrogen bonds and inter­molecular π–π stacking inter­actions between adjacent bpy ligands [centroid–centroid distance = 3.617 (2) Å] in the structure.

## Related literature

For Cl atoms acting as the bridging anions in transition metal complexes in multi-dimensional mol­ecule-based magnetic materials, see: Jian *et al.* (2005 ▶). For related structures, see: Leznoff *et al.* (2003 ▶); Liu *et al.*(2004 ▶); Puschmann *et al.* (2001 ▶).
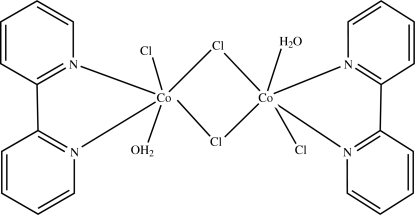


## Experimental

### 

#### Crystal data

[Co_2_Cl_4_(C_10_H_8_N_2_)_2_(H_2_O)_2_]
*M* *_r_* = 608.06
Monoclinic, 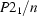

*a* = 11.2939 (10) Å
*b* = 6.8969 (6) Å
*c* = 15.1339 (13) Å
β = 91.958 (3)°
*V* = 1178.14 (18) Å^3^
*Z* = 2
Mo *K*α radiation
μ = 1.89 mm^−1^
*T* = 293 K
0.26 × 0.20 × 0.20 mm

#### Data collection

Rigaku SCXmini diffractometer
Absorption correction: multi-scan (*CrystalClear*; Rigaku, 2005 ▶) *T*_min_ = 0.641, *T*_max_ = 0.688
11707 measured reflections
2693 independent reflections
2149 reflections with *I* > 2σ(*I*)
*R*_int_ = 0.054

#### Refinement

*R*[*F* ^2^ > 2σ(*F* ^2^)] = 0.037
*wR*(*F* ^2^) = 0.085
*S* = 1.03
2693 reflections
145 parameters
H-atom parameters constrained
Δρ_max_ = 0.41 e Å^−3^
Δρ_min_ = −0.49 e Å^−3^

### 

Data collection: *CrystalClear* (Rigaku, 2005 ▶); cell refinement: *CrystalClear*; data reduction: *CrystalClear*; program(s) used to solve structure: *SHELXS97* (Sheldrick, 2008 ▶); program(s) used to refine structure: *SHELXL97* (Sheldrick, 2008 ▶); molecular graphics: *SHELXTL* (Sheldrick, 2008 ▶); software used to prepare material for publication: *SHELXTL*.

## Supplementary Material

Crystal structure: contains datablocks I, global. DOI: 10.1107/S1600536809027846/at2843sup1.cif

Structure factors: contains datablocks I. DOI: 10.1107/S1600536809027846/at2843Isup2.hkl

Additional supplementary materials:  crystallographic information; 3D view; checkCIF report

## Figures and Tables

**Table 1:** Hydrogen-bond geometry (Å, °)

*D*—H⋯*A*	*D*—H	H⋯*A*	*D*⋯*A*	*D*—H⋯*A*
O1—H1*B*⋯Cl1^i^	0.82	2.81	3.407 (2)	132
O1—H1*C*⋯Cl1^ii^	0.85	2.39	3.213 (2)	162

Symmetry codes: (i) 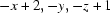; (ii) 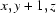.

**Fig. 1.**
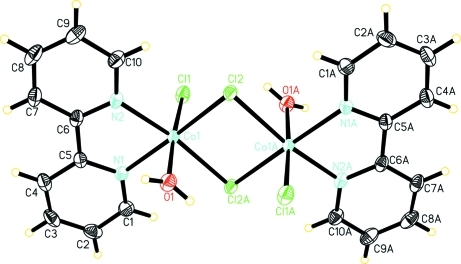
The molecular structure of the title compound with the atom-numbering scheme and all hydrogen atoms. Displacement ellipsoids are drawn at the 30% probability level. [Symmetry code A: 2 - x, -y, 1 - z]

**Fig. 2.**
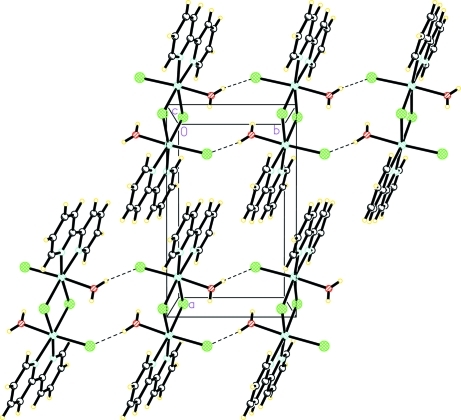
Crystal packing of the compound (I). Hydrogen bonds are shown as dashed lines.

## supplementary crystallographic information

### Comment

In the study of multidimensional molecule-based magnetic materials and other areas, the Cl atoms acting as the bridging anions has frequently been used to bridge transition metal complexes (Jian *et al.*, 2005). Many such compounds have been reported (Leznoff *et al.*, 2003; Liu *et al., *2004; Puschmann *et al.*, 2001). Herein, we reported the structure of the title Co^II^ compound (I).

The two Co atoms are bridged by two Cl anions into a four-membered Co_2_Cl_2_ ring. The Co atom is six-coordinated by three Cl atoms, one water molecules and one 2,2'-bipy ligand in an octahedral geometry. The molecule has an inversion symmetry (Fig. 1). In the crystal structure, the intermolecular O—H···Cl hydrogen bonds connect the molecules of (I) into a one-dimensional chain structure. There are π-π stacking interactions between adjacent bpy (2,2'-bipyridine) ligands, where the centroid–centroid separations are 3.617 (2) Å. π-π stacking interaction existing in every two O—H···Cl hydrogen bonds chains, and forming pairs of complex molecules into a two-dimensional structure (Fig. 2). A supramolecular network structure is consolidated by π-π stacking and hydrogen bonds.

### Experimental

All chemicals used (reagent grade) were commercially available. To a 10 ml MeCN solution of cobalt dichloride hexahydrate (0.0238 g, 0.1 mmol), a 4 ml CH_2_Cl_2_ solution of 2,2'-bipyridine (0.0156 g, 0.1 mmol) was added dropwise with stirring. The resulting solution was continuously stirred for about 30 min, and then filtered. The filtrate was slowly evaporated at room temperature over several days, and colourless prism crystals suitable for X-ray analysis were obtained.

### Refinement

The positions of the C-bound H atoms were calculated geometrically and refined using a riding model with C-H = 0.93Å and *U*_iso_(H) = 1.2*U*_eq_(C). The positional parameters for the H atoms of the water molecule were placed geometrically and refined with a fixed *U*_iso_ of 0.05.

### Crystal data

**Table:**

[Co_2_Cl_4_(C_10_H_8_N_2_)_2_(H_2_O)_2_]	*F*(000) = 612
*M**_r_* = 608.06	*D*_x_ = 1.714 Mg m^−^^3^
Monoclinic, *P*2_1_/*n*	Mo *K*α radiation, λ = 0.71073 Å
Hall symbol: -P 2yn	Cell parameters from 10382 reflections
*a* = 11.2939 (10) Å	θ = 3.2–27.7°
*b* = 6.8969 (6) Å	µ = 1.89 mm^−^^1^
*c* = 15.1339 (13) Å	*T* = 293 K
β = 91.958 (3)°	Prism, colourless
*V* = 1178.14 (18) Å^3^	0.26 × 0.20 × 0.20 mm
*Z* = 2	

### Data collection

**Table:**

Rigaku SCXmini diffractometer	2693 independent reflections
Radiation source: fine-focus sealed tube	2149 reflections with *I* > 2σ(*I*)
graphite	*R*_int_ = 0.054
Detector resolution: 8.192 pixels mm^-1^	θ_max_ = 27.5°, θ_min_ = 3.3°
ω scans	*h* = −14→14
Absorption correction: multi-scan (*CrystalClear*; Rigaku, 2005)	*k* = −8→8
*T*_min_ = 0.641, *T*_max_ = 0.688	*l* = −19→19
11707 measured reflections	

### Refinement

**Table:**

Refinement on *F*^2^	Primary atom site location: structure-invariant direct methods
Least-squares matrix: full	Secondary atom site location: difference Fourier map
*R*[*F*^2^ > 2σ(*F*^2^)] = 0.037	Hydrogen site location: inferred from neighbouring sites
*wR*(*F*^2^) = 0.085	H-atom parameters constrained
*S* = 1.03	*w* = 1/[σ^2^(*F*_o_^2^) + (0.0346*P*)^2^ + 0.3472*P*] where *P* = (*F*_o_^2^ + 2*F*_c_^2^)/3
2693 reflections	(Δ/σ)_max_ = 0.001
145 parameters	Δρ_max_ = 0.41 e Å^−^^3^
0 restraints	Δρ_min_ = −0.49 e Å^−^^3^

### Special details

**Table:**

Geometry. All e.s.d.'s (except the e.s.d. in the dihedral angle between two l.s. planes) are estimated using the full covariance matrix. The cell e.s.d.'s are taken into account individually in the estimation of e.s.d.'s in distances, angles and torsion angles; correlations between e.s.d.'s in cell parameters are only used when they are defined by crystal symmetry. An approximate (isotropic) treatment of cell e.s.d.'s is used for estimating e.s.d.'s involving l.s. planes.
Refinement. Refinement of *F*^2^ against ALL reflections. The weighted *R*-factor *wR* and goodness of fit *S* are based on *F*^2^, conventional *R*-factors *R* are based on *F*, with *F* set to zero for negative *F*^2^. The threshold expression of *F*^2^ > σ(*F*^2^) is used only for calculating *R*-factors(gt) *etc*. and is not relevant to the choice of reflections for refinement. *R*-factors based on *F*^2^ are statistically about twice as large as those based on *F*, and *R*- factors based on ALL data will be even larger.

### Fractional atomic coordinates and isotropic or equivalent isotropic displacement parameters (Å^2^)

**Table:**

	*x*	*y*	*z*	*U*_iso_*/*U*_eq_	
Co1	0.84715 (3)	0.03942 (5)	0.50354 (2)	0.02938 (12)	
O1	0.90322 (17)	0.3279 (3)	0.55130 (12)	0.0420 (5)	
H1B	0.9629	0.3171	0.5833	0.050*	
H1C	0.8639	0.4280	0.5357	0.050*	
N1	0.71222 (19)	0.0653 (3)	0.59523 (14)	0.0328 (5)	
N2	0.70852 (19)	0.1700 (3)	0.42759 (14)	0.0340 (5)	
C1	0.7206 (3)	0.0099 (4)	0.67984 (19)	0.0418 (7)	
H1A	0.7894	−0.0524	0.6999	0.050*	
C2	0.6322 (3)	0.0407 (5)	0.7389 (2)	0.0504 (8)	
H2A	0.6418	0.0035	0.7977	0.060*	
C3	0.5291 (3)	0.1284 (4)	0.7079 (2)	0.0500 (8)	
H3A	0.4673	0.1493	0.7459	0.060*	
C4	0.5178 (3)	0.1848 (4)	0.6209 (2)	0.0441 (7)	
H4A	0.4485	0.2437	0.5995	0.053*	
C5	0.6114 (2)	0.1524 (4)	0.56541 (18)	0.0325 (6)	
C6	0.6095 (2)	0.2104 (4)	0.47093 (19)	0.0345 (6)	
C7	0.5126 (3)	0.2985 (4)	0.4275 (2)	0.0430 (7)	
H7A	0.4448	0.3272	0.4581	0.052*	
C8	0.5184 (3)	0.3423 (4)	0.3396 (2)	0.0513 (9)	
H8A	0.4543	0.4011	0.3102	0.062*	
C9	0.6192 (3)	0.2994 (4)	0.2947 (2)	0.0493 (8)	
H9A	0.6242	0.3275	0.2348	0.059*	
C10	0.7123 (3)	0.2134 (4)	0.34111 (19)	0.0430 (7)	
H10A	0.7807	0.1842	0.3113	0.052*	
Cl1	0.79648 (6)	−0.29082 (10)	0.45617 (5)	0.0445 (2)	
Cl2	0.99818 (6)	0.07599 (10)	0.39243 (4)	0.03617 (17)	

### Atomic displacement parameters (Å^2^)

**Table:**

	*U*^11^	*U*^22^	*U*^33^	*U*^12^	*U*^13^	*U*^23^
Co1	0.0223 (2)	0.0359 (2)	0.0300 (2)	0.00151 (15)	0.00089 (14)	0.00302 (16)
O1	0.0374 (11)	0.0386 (11)	0.0496 (12)	−0.0004 (9)	−0.0023 (9)	−0.0006 (10)
N1	0.0269 (12)	0.0365 (12)	0.0349 (12)	−0.0016 (10)	0.0014 (9)	−0.0011 (10)
N2	0.0264 (12)	0.0348 (12)	0.0405 (13)	−0.0018 (9)	−0.0020 (10)	0.0042 (10)
C1	0.0373 (17)	0.0510 (17)	0.0373 (15)	−0.0046 (13)	0.0049 (13)	0.0032 (13)
C2	0.052 (2)	0.063 (2)	0.0364 (16)	−0.0128 (17)	0.0123 (15)	−0.0067 (15)
C3	0.0406 (18)	0.0510 (19)	0.060 (2)	−0.0077 (15)	0.0227 (15)	−0.0188 (16)
C4	0.0323 (16)	0.0383 (16)	0.062 (2)	−0.0014 (13)	0.0101 (14)	−0.0134 (15)
C5	0.0263 (14)	0.0254 (13)	0.0459 (15)	−0.0025 (11)	0.0025 (12)	−0.0061 (12)
C6	0.0268 (14)	0.0259 (13)	0.0503 (16)	−0.0012 (11)	−0.0052 (12)	−0.0037 (12)
C7	0.0308 (15)	0.0313 (15)	0.066 (2)	0.0051 (12)	−0.0099 (14)	−0.0088 (14)
C8	0.051 (2)	0.0340 (16)	0.066 (2)	0.0049 (14)	−0.0279 (17)	0.0019 (15)
C9	0.051 (2)	0.0462 (18)	0.0489 (18)	−0.0039 (15)	−0.0170 (15)	0.0133 (15)
C10	0.0374 (16)	0.0493 (18)	0.0420 (16)	−0.0032 (13)	−0.0043 (13)	0.0103 (14)
Cl1	0.0343 (4)	0.0364 (4)	0.0624 (5)	−0.0001 (3)	−0.0050 (3)	−0.0031 (3)
Cl2	0.0264 (3)	0.0535 (4)	0.0286 (3)	0.0013 (3)	0.0003 (3)	0.0078 (3)

### Geometric parameters (Å, °)

**Table:**

Co1—N1	2.103 (2)	C2—H2A	0.9300
Co1—N2	2.112 (2)	C3—C4	1.375 (4)
Co1—O1	2.2021 (18)	C3—H3A	0.9300
Co1—Cl2^i^	2.4445 (7)	C4—C5	1.391 (4)
Co1—Cl2	2.4488 (7)	C4—H4A	0.9300
Co1—Cl1	2.4497 (8)	C5—C6	1.484 (4)
O1—H1B	0.8200	C6—C7	1.396 (4)
O1—H1C	0.8500	C7—C8	1.368 (4)
N1—C1	1.336 (3)	C7—H7A	0.9300
N1—C5	1.351 (3)	C8—C9	1.377 (5)
N2—C10	1.345 (3)	C8—H8A	0.9300
N2—C6	1.345 (3)	C9—C10	1.379 (4)
C1—C2	1.378 (4)	C9—H9A	0.9300
C1—H1A	0.9300	C10—H10A	0.9300
C2—C3	1.380 (5)	Cl2—Co1^i^	2.4445 (7)
			
N1—Co1—N2	77.44 (8)	C1—C2—H2A	121.0
N1—Co1—O1	85.04 (8)	C3—C2—H2A	121.0
N2—Co1—O1	89.59 (8)	C4—C3—C2	119.9 (3)
N1—Co1—Cl2^i^	96.93 (6)	C4—C3—H3A	120.1
N2—Co1—Cl2^i^	171.68 (7)	C2—C3—H3A	120.1
O1—Co1—Cl2^i^	83.78 (5)	C3—C4—C5	119.0 (3)
N1—Co1—Cl2	169.00 (6)	C3—C4—H4A	120.5
N2—Co1—Cl2	95.92 (6)	C5—C4—H4A	120.5
O1—Co1—Cl2	86.16 (5)	N1—C5—C4	121.3 (3)
Cl2^i^—Co1—Cl2	88.66 (2)	N1—C5—C6	115.2 (2)
N1—Co1—Cl1	96.01 (6)	C4—C5—C6	123.5 (3)
N2—Co1—Cl1	94.35 (6)	N2—C6—C7	120.8 (3)
O1—Co1—Cl1	176.05 (5)	N2—C6—C5	115.4 (2)
Cl2^i^—Co1—Cl1	92.31 (3)	C7—C6—C5	123.8 (3)
Cl2—Co1—Cl1	93.21 (3)	C8—C7—C6	119.4 (3)
Co1—O1—H1B	109.5	C8—C7—H7A	120.3
Co1—O1—H1C	120.1	C6—C7—H7A	120.3
H1B—O1—H1C	130.4	C7—C8—C9	120.0 (3)
C1—N1—C5	118.6 (2)	C7—C8—H8A	120.0
C1—N1—Co1	125.36 (19)	C9—C8—H8A	120.0
C5—N1—Co1	116.00 (17)	C8—C9—C10	118.0 (3)
C10—N2—C6	118.9 (2)	C8—C9—H9A	121.0
C10—N2—Co1	125.24 (19)	C10—C9—H9A	121.0
C6—N2—Co1	115.86 (18)	N2—C10—C9	122.9 (3)
N1—C1—C2	123.2 (3)	N2—C10—H10A	118.6
N1—C1—H1A	118.4	C9—C10—H10A	118.6
C2—C1—H1A	118.4	Co1^i^—Cl2—Co1	91.34 (2)
C1—C2—C3	118.0 (3)		
			
N2—Co1—N1—C1	−179.5 (2)	C1—N1—C5—C6	179.7 (2)
O1—Co1—N1—C1	−88.8 (2)	Co1—N1—C5—C6	2.2 (3)
Cl2^i^—Co1—N1—C1	−5.7 (2)	C3—C4—C5—N1	0.6 (4)
Cl2—Co1—N1—C1	−125.8 (3)	C3—C4—C5—C6	−178.9 (3)
Cl1—Co1—N1—C1	87.3 (2)	C10—N2—C6—C7	−0.9 (4)
N2—Co1—N1—C5	−2.25 (17)	Co1—N2—C6—C7	179.38 (19)
O1—Co1—N1—C5	88.43 (18)	C10—N2—C6—C5	178.2 (2)
Cl2^i^—Co1—N1—C5	171.53 (17)	Co1—N2—C6—C5	−1.5 (3)
Cl2—Co1—N1—C5	51.4 (4)	N1—C5—C6—N2	−0.5 (3)
Cl1—Co1—N1—C5	−95.39 (17)	C4—C5—C6—N2	179.1 (2)
N1—Co1—N2—C10	−177.7 (2)	N1—C5—C6—C7	178.7 (2)
O1—Co1—N2—C10	97.3 (2)	C4—C5—C6—C7	−1.8 (4)
Cl2—Co1—N2—C10	11.2 (2)	N2—C6—C7—C8	0.7 (4)
Cl1—Co1—N2—C10	−82.5 (2)	C5—C6—C7—C8	−178.4 (3)
N1—Co1—N2—C6	1.99 (18)	C6—C7—C8—C9	0.0 (4)
O1—Co1—N2—C6	−83.02 (18)	C7—C8—C9—C10	−0.4 (4)
Cl2—Co1—N2—C6	−169.12 (17)	C6—N2—C10—C9	0.5 (4)
Cl1—Co1—N2—C6	97.19 (18)	Co1—N2—C10—C9	−179.8 (2)
C5—N1—C1—C2	−1.3 (4)	C8—C9—C10—N2	0.1 (4)
Co1—N1—C1—C2	175.9 (2)	N1—Co1—Cl2—Co1^i^	120.8 (3)
N1—C1—C2—C3	1.8 (5)	N2—Co1—Cl2—Co1^i^	173.04 (6)
C1—C2—C3—C4	−1.0 (5)	O1—Co1—Cl2—Co1^i^	83.85 (5)
C2—C3—C4—C5	−0.1 (4)	Cl2^i^—Co1—Cl2—Co1^i^	0.0
C1—N1—C5—C4	0.1 (4)	Cl1—Co1—Cl2—Co1^i^	−92.24 (3)
Co1—N1—C5—C4	−177.4 (2)		

Symmetry codes: (i) −*x*+2, −*y*, −*z*+1.

### Hydrogen-bond geometry (Å, °)

**Table:**

*D*—H···*A*	*D*—H	H···*A*	*D*···*A*	*D*—H···*A*
O1—H1B···Cl1^i^	0.82	2.81	3.407 (2)	132
O1—H1C···Cl1^ii^	0.85	2.39	3.213 (2)	162

Symmetry codes: (i) −*x*+2, −*y*, −*z*+1; (ii) *x*, *y*+1, *z*.
